# SMART-RNA-Metavirome: a practical RNA metavirome platform compatible with high-throughput sequencing of both short and long reads

**DOI:** 10.1186/s40249-025-01371-z

**Published:** 2025-10-10

**Authors:** Xiaohua Liu, Ziyao Li, Xiang Guo, Liu Ge, Minling Hu, Qing He, Xiaoqing Zhang, Ziqing Feng, Yuji Wang, Lingzhai Zhao, Shu Zeng, Wenwen Ren, Haiyang Chen, Chunmei Wang, Rangke Wu, Wei Zhao, Fuchun Zhang, Xiao-Guang Chen, Xiaohong Zhou

**Affiliations:** 1 Institute of Tropical Medicine, Department of Pathogen Biology, School of Public Health, Southern Medical University; Guangdong Provincial Key Laboratory of Tropical Disease Research; Key Laboratory of Prevention and Control for Emerging Infectious Diseases of Guangdong Higher Institutes; Key Laboratory of Infectious Diseases Research in South China, Ministry of Education, Guangzhou, 510515 Guangdong China; 2 The Fifth Affiliated Hospital of Southern Medical University, Guangzhou, 510900 Guangdong China; 3 School of Basic Medical Sciences, Henan University, Kaifeng, 475001 Henan China; 4 School of Basic Medicine and Forensics, Hangzhou Medical College, Hangzhou, 310000 Zhejiang China; 5 Institute of Infectious Diseases, Guangzhou Eighth People’s Hospital, Guangzhou Medical University, Guangzhou, 510440 Guangdong China; 6 School of Foreign Studies, Southern Medical University, Guangzhou, 510515 Guangdong China; 7 BSL-3 Laboratory (Guangdong), School of Public Health, Southern Medical University, Guangzhou, 510515 Guangdong China

**Keywords:** SMART-RNA-Metavirome, Next-generation sequencing, Third-generation sequencing, Arboviruses, *Aedes albopictus*

## Abstract

**Background:**

The RNA virosphere's extensive diversity and its role in emerging infectious diseases underscore the importance of non-targeted sequencing for identifying unknown or rare pathogens, including co-infections. However, enriching low-abundance viral sequences in RNA metaviromics, particularly in the preparation of cDNA libraries and their compatibility with next-generation sequencing (NGS) and third-generation sequencing (TGS), remains challenging. Therefore, our objective is to develop and systematically assess a practical RNA metavirome methodology specifically tailored for the enrichment of low-abundance viral sequences within samples.

**Methods:**

We developed the SMART-RNA-Metavirome platform, integrating SMART-9n library preparation with NGS and TGS technologies. Total RNA was extracted from two field-collected wild *Aedes albopictus* pools, along with one laboratory-infected *Ae. albopictus* pool harboring dengue virus (DENV). This RNA was subjected to reverse transcription using both this optimized protocol and random primer-based methods, followed by high-throughput sequencing on Illumina, Oxford Nanopore, and QitanTech Nanopore technologies. Welch's t-test was employed for comparative analysis of the subsequent RNA metavirome data, specifically to evaluate differences in viral species composition and abundance of viral reads between experimental groups. Furthermore, the effectiveness of this platform was systematically validated via RT-qPCR and SMART-RNA-Metavirome-based Oxford Nanopore sequencing across multiple sample types, including mosquito specimens from DENV-infected *Ae. albopictus*, serum samples from dengue patients and viral isolates of Japanese encephalitis virus (JEV) and Zika virus (ZIKV).

**Results:**

The SMART-RNA-Metavirome platform has been systematically validated to excel in enriching the composition and diversity of the RNA virome (*P* = 0.04), providing sufficient coverage for the complete reconstruction of viral genomes. When employed in the detection of DENV-infected *Ae. albopictus*, clinical serum samples, and viral isolates of JEV and ZIKV, this technique exhibits a robust correlation with RT-qPCR (*r*^2^ > 0.95). Notably, it demonstrates exceptional sensitivity, ensuring sufficient coverage even in samples of DENV-infected *Ae. albopictus* with a Ct-value of 35.3, attaining an impressive 99.88% genome coverage. Furthermore, this platform possesses the capability to identify virus species and determine their serotypes.

**Conclusions:**

In our study, the SMART-RNA-Metavirome platform outperforms traditional methods, enriching RNA virome composition and diversity, enabling practical compatibility with both NGS and TGS technologies. It demonstrates significant proficiency in detecting both known and unknown arboviruses, even in low-titer samples such as those from wild mosquitoes and clinical sera. This platform facilitates comprehensive monitoring, risk assessment, and early warning of RNA virus transmissions, enhancing our understanding of RNA virome diversity and ecological patterns.

**Graphical abstract:**

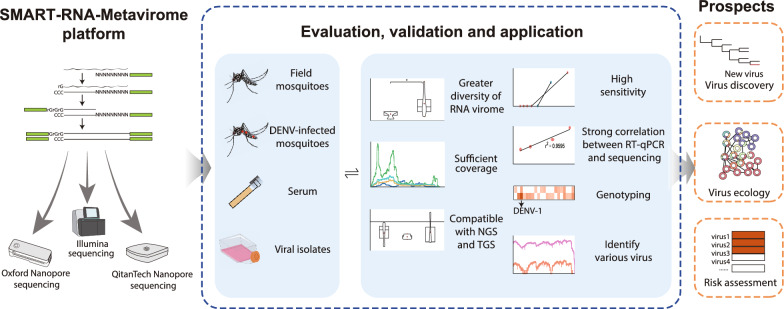

**Supplementary Information:**

The online version contains supplementary material available at 10.1186/s40249-025-01371-z.

## Background

RNA viruses play a significant role as etiological agents in both human and veterinary infectious diseases, including those caused by dengue virus (DENV) [1], Zika virus (ZIKV) [2], Japanese encephalitis virus (JEV) [3], Ebola virus (EBOV) [4], and severe acute respiratory syndrome coronavirus 2 (SARS-CoV-2) [5]. They account for 44% of emerging and re-emerging infections, posing substantial threats to global health and the economy [6]. The continuous genetic variation of these viruses presents a formidable challenge for the molecular diagnosis of viral diseases. Probing into these diverse pathogens through reverse microbial etiology can proactively mitigate outbreaks of infectious diseases [7].

Traditionally, isolating and discovering novel viruses has primarily involved culturing them in suitable cell lines followed by microscopic examination [8]. However, these conventional methods have inherent limitations in detecting a wide range of viruses, largely due to the difficulties in culturing viruses in vitro and their dependence on specific environmental conditions for viability. Molecular biological techniques, such as reverse transcription-polymerase chain reaction (RT-PCR) and serological tests, require prior knowledge of the infectious agent [9], thereby limiting their applicability in viral discovery efforts. In contrast, metaviromics, a pivotal technology in reverse microbial etiology, leverages high-throughput sequencing and bioinformatics analysis. This approach has become indispensable in the prevention and control of emerging and re-emerging infections, offering targeted and untargeted means of amplifying and sequencing the entire nucleic acid content.

Existing high-throughput sequencing technologies include next-generation sequencing (NGS) and third-generation sequencing (TGS). NGS has been widely adopted for metavirome detection, effectively enabling the identification and characterization of viruses in plants [10], invertebrates [11–13], vertebrates [14], and aquatic environments. However, NGS has led to the generation of substantial volumes of raw data consisting of short reads, which necessitates the utilization of high-performance computers and extensive computational analysis for assembly. Additionally, the application of NGS is still limited by the extended sample-to-answer turnaround times and high costs. The advent of TGS, in the case of Nanopore sequencing, has enabled the generation of long reads and the possibility of analyzing data in real-time. To date, Nanopore sequencing has demonstrated remarkable success in diagnosing infectious diseases through targeted PCR-based amplicon or capture-based probe hybridization for viruses, such as ZIKV [15], DENV-2 [16], chikungunya virus (CHIKV) [17], yellow fever virus (YFV) [18], and EBOV [19]. But it requires prior knowledge of the viruses. Therefore, its effective application in metavirome research remains challenging.

Compared to targeted sequencing, non-targeted sequencing operates independently of prior knowledge about the target pathogen, rendering it a valuable tool for detecting unknown pathogens, rare pathogens, clinical specimens with undetermined etiologies, and co-infections. Since the performance of both NGS and TGS depends on the DNA-based sequencing instruments employed, enhancing the preparation of cDNA libraries from RNA is a crucial step in the RNA metavirome technique. During the library preparation process, both specific viral sequences and non-specific host sequences undergo simultaneous amplification; however, the enrichment of specific viral sequences poses a significant challenge due to their extremely low proportion relative to the total sequences. To overcome this challenge, various approaches have been developed for cDNA library preparation, aimed at either enriching viral-specific sequences or depleting ribosomal RNA (rRNA). These methods encompass sequence-independent single primer amplification (SISPA) [20], rolling circle amplification (RCA) [21] and switching mechanism at the 5′ end of the RNA transcript (SMART) [22], etc.

SMART, initially proposed by Zhu et al. [22], offers a streamlined and rapid approach for the reverse transcription of RNA into cDNA. This technique relies on oligo (dT) priming to selectively target polyadenylated mRNA molecules. Currently, it is extensively employed in single-cell sequencing, with the development of several commercial kits based on this methodology (e.g., SMARTer Ultra Low RNA Kit; Clontech). In 2021, Ingra M. Claro proposed SMART-9n to amplify non-poly(A) tailing RNA, by substituting the poly(T) primer with a 9N primer [23]. The SMART-9N technology employs a 9N primer containing nine degenerate bases to non-preferentially target all RNA sequences for first-strand cDNA synthesis. Upon encountering the 5′ cap structure of RNA, the reverse transcriptase adds three cytosines to the 3′ end of the cDNA. This facilitates template-switching activity, enabling second-strand synthesis to be initiated from a template-switching oligonucleotide. Consequently, ribosomal RNAs (rRNAs) lacking cap structures will not be reverse transcribed, effectively reducing background nucleic acid interference. This represents a critical advantage unattainable with random primer-based methods, particularly for samples where rRNA cannot be removed by commercial depletion kits. Furthermore, in comparison to approaches relying on random primers, this particular process generates longer cDNA fragments. This attribute endows SMART-9N with intrinsic compatibility with TGS. Moreover, the specific primers incorporated during reverse transcription also enable PCR amplification with reduced amplification bias compared to random primer-based methods. Subsequently, the initial assessment of SMART-9n's potential was conducted using metavirome methods specifically tailored for the targeted detection of ZIKV, YFV, and SARS-CoV-2 in cultured viral samples [23]. In the present study, we have devised a non-targeted SMART-RNA-Metavirome platform, representing a practical RNA metavirome technique that is compatible with high-throughput sequencing of both short and long reads. This methodology underwent comprehensive systematic evaluation and subsequent validation using infected mosquito samples, clinical serum specimens, and various viral isolates.

## Methods

### Field *Ae. albopictus* collection

In September 2022, handheld portable collectors were utilized to sample the wild populations of *Ae. albopictus* from designated investigation sites in Guangzhou (GZ) and Zhongshan (ZS), both located within Guangdong Province, China. Mosquitoes were collected and preserved in vacuum bottles containing ice and then transported to the laboratory. After being frozen at − 20 °C for a duration of 15 min, the mosquitoes underwent morphological identification to determine their species using the taxonomic key provided by Lu et al. [24, 25]. Subsequently, the specimens were subjected to DNA sequencing of the cytochrome coxidase subunit I mitochondrial gene (*cox*I) for further genetic analysis. Following these procedures, the mosquitoes were assigned to pools, each comprising ten individuals. These pools were categorized based on both species classification and geographic origins.

### Mosquito strain and DENV strain

The Foshan strain of *Ae. albopictus* was obtained from the Center for Disease Control and Prevention of Guangdong Province, China, where it has been maintained in culture since 1981. Mosquitoes were reared at a temperature of 28 ± 1 °C with a relative humidity of 70–80%, under a light/dark cycle of 16/8 h. The DENV-1 strain N46V (GenBank Accession No. KX458014.1) was isolated from serum samples collected during dengue outbreaks in 2014 in Guangdong Province, China.

### Oral infection of DENV-1 in mosquitoes

The DENV-1 strain N46V was propagated in C6/36 cells and quantified using RT-qPCR. The cell supernatant was harvested and mixed with sterile defibrinated sheep blood at a ratio of 2:1, with the mixture then incubated at 37 °C for 5 min. Five days post-emergence, the female mosquitoes were selected and subjected to a 12-h starvation period. These hungry female mosquitoes were then fed on the prepared blood meal using a blood reservoir for 45 min. The mosquitoes were then anesthetized briefly by freezing at − 20 °C for 20 s. These fully engorged mosquitoes were individually transferred to cages and supplied with 10% glucose water and maintained under standardized insectary conditions (28 ± 1 °C, 70–80% relative humidity, and a 16 h:8 h light–dark photoperiod) for a period of 14 days.

### Nucleic acid extraction

Total RNA was extracted from the mosquito samples including DENV-infected *Ae. albopictus* (D1) and the wild populations of *Ae. albopictus* sampled in Guangzhou (GZ) and Zhongshan (ZS), with each pool consisting of 10 mosquitoes. Prior to homogenization with a tissue grinder in phosphate buffered saline (PBS, Cat. No.C10010500BT, Gibco, Grand Island, NY, USA), the sampling pools of *Ae. albopictus* were rinsed with the same PBS solution. Total RNA was extracted using AG RNAex Pro Reagent (Cat. No. AG21102, Accurate Biology, Hunan, China) according to the manufacturer’s instructions. The quantity and quality of the extracted RNA were evaluated using a NanoDrop 2000 spectrophotometer (Thermo Fisher Scientific, Waltham, MA, USA).

### cDNA synthesis using SMART-RNA-Metavirome

For the first strand cDNA synthesis, total RNA (1 µg) from each sample was mixed with deoxyribonucleotide triphosphates (dNTPs) mixture (1 µl, 10 mmol/L, Cat. No. N0447V, New England BioLabs, Beverly, MA, USA), 9N primer (1 µl, 2 µmol/L, sequence: AAGCA GTGGT ATCAA CGCAG AGTAC NNNNN NNNN), and nuclease-free water to attain a final volume of 12 µl. The mixture was incubated at 65 °C for 5 min. For the second strand cDNA synthesis, the resultant product (12 µl) was mixed with 5 × RTase Reaction Buffer III (4 µl, Cat. No. AG11617, Accurate Biology, Hunan, China), DTT (1 µl, 0.1 mol/L Cat. No. 2141347, Invitrogen), RNase OUT (1µl, Cat. No. 10777019, Thermo Fisher Scientific, Waltham, MA, USA), SSP primer [1 µl, 2 µmol/L, sequence: GCTAA TCATT GCAAG CAGTG GTATC AACGC AGAGT ACATrGrGrG, in which the rGrGrG represents three guanine ribonucleotides, with the last rG being Locked Nucleic Acid (LNA)-modified], and Evo M-MLV III RTase (1 µl, 100U/µl, Cat. No. AG11617, Accurate Biology, Hunan, China). The mixture was then incubated at 42 °C for 90 min, followed by a 10-min incubation at 70 °C.

For PCR amplification, cDNA products (5 µl) were mixed with dNTPs (1µl, 10 mmol/L), NEB PCR primer (2 µl, 20 µmol/L, sequence: AAGCA GTGGT ATCAAC GCAGA GT), Q5 DNA polymerase (0.5 µl, Cat.No. M0491V, New England BioLabs, Beverly, MA, USA), Q5 reaction buffer (10 µl), and nuclease-free water (31.5 µl). The PCR protocol included an initial cycle at 98 °C for 45 s, followed by 30 cycles of 98 °C for 15 s, 62 °C for 15 s, and 65 °C for 5 min, with a final extension at 65 °C for 10 min. The amplified products were purified using AMPure XP beads (Cat. No. A63881, Beckman Coulter, Brea, CA, USA) at a ratio of 1:1 and quantified using the Qubit dsDNA Broad Range fluorometric assay (Cat. No. Q32854, Thermo Fisher Scientific, Waltham, MA, USA) on the Qubit 4.0 instrument (Thermo Fisher Scientific, Waltham, MA, USA), according to the manufacturer’s instructions.

### Library preparation and sequencing

Random Primer Library Combined with Illumina Sequencing: Sequencing libraries were generated using NEB Next® Ultra™ DNA Library Prep Kit for Illumina® (New England Biolabs, MA, USA) following manufacturer's recommendations and index codes were added. The library quality was assessed on the Qubit® dsDNA HS Assay Kit (Thermo Fisher Scientific, Waltham, MA, USA) and Agilent 4200 (Agilent Technologies, Santa Clara, CA, USA) system. At last, the library was sequenced on an Illumina Novaseq 6000 and 150 bp paired-end reads were generated.

SMART-RNA-Metavirome Library-based Illumina Sequencing: Sequencing libraries were prepared using the NEB Next® Ultra™ DNA Library Prep Kit for Illumina (New England Biolabs, MA, USA) following the manufacturer's guidelines. Index codes were incorporated during this process to facilitate multiplex sequencing. The quality of the prepared libraries was evaluated using the Qubit® dsDNA HS Assay Kit (Thermo Fisher Scientific, Waltham, MA, USA) for quantitative assessment and Agilent 4200 TapeStation System (Agilent Technologies, Santa Clara, CA, USA) for size distribution and purity analysis. Finally, sequencing was performed on an Illumina Novaseq 6000 platform, generating high-quality 150 bp paired-end reads for further genomic analysis.

SMART-RNA-Metavirome Library-based Oxford Nanopore Sequencing: Sequencing libraries were prepared using the Oxford Nanopore SQK-LSK109 kit and Native Barcoding Expansion 13-24(EXP-NBD114) (ONT, Oxford, UK), following the manufacturer's instructions. Subsequently, the libraries were loaded onto a FLO-MIN106 flow cell, and then inserted into the MinION sequencing device (ONT, Oxford, UK). Sequencing was conducted using the MinKNOW software for seamless operation and data acquisition.

SMART-RNA-Metavirome Library-based QitanTech Nanopore Sequencing: Sequencing libraries were prepared by Qitan’s technical team using the QDL-E v2.0 reagent kit (Qitan Technology Co., Chengdu, Sichuan, China) according to the manufacturer’s guidelines. The prepared libraries were loaded onto a QCell-384-P V2.0 sequencing flow cell, and then assembled onto a QNome-3841 sequencer. This sequencer was connected to a standard personal computer via a USB cable for data transfer and monitoring. Sequencing was performed using the stand-alone QPreasy-NF v1.3.12 (https://www.qitantech.com/) software, enabling real-time basecalling on a GPU-equipped computer. This basecalling process leveraged a deep neural network algorithm-based basecaller for accurate and efficient sequence determination.

### Sequencing data analysis

Illumina Sequencing Data Analysis: The initial raw sequencing reads underwent adapter and quality trimming using Trimmomatic v0.39 (https://www.plabipd.de/trimmomatic_main.html) [26]. To maintain comparability among different sequencing methodologies, one of the paired-end sequencing reads was normalized to ensure an equivalent number of sequencing bases using SeqKit v2.4.0 (https://github.com/shenwei356/seqkit) [27]. This normalization accounts for the fact that TGS typically provides single-end sequencing reads, but NGS provides paired-end sequencing reads, and provides more but shorter sequencing reads than TGS. For SMART-RNA-Metavirome-based Illumina sequencing, the primers of the SMART-RNA-Metavirome (GTACTCTGCGTTGATACCACTGCTT for 5′ primer and AAGCAGTGGTATCAACGCAGAGTACATGGG for 3′ primer, or CCCATGTACTCTGCGTTGATACCACTGCTT for 5′ primer and AAGCAGTGGTATCAACGCAGAGTAC for 3′ primer) were cut using Cutadapt v1.18 (https://cutadapt.readthedocs.io/) [28]. The curated reads were compared against the nr database (updated in 2022), which was obtained from the National Center for Biotechnology Information (NCBI). This comparison was performed using Diamond blastx v0.9.21 (https://github.com/bbuchfink/diamond), with a cut-off E-value of 1 × 10^–5^ [29]. The top blast hit was retained using custom-written code in R v4.2 (https://www.r-project.org/) and then their taxonomy was obtained using TaxonKit v0.14.1 (https://github.com/shenwei356/taxonkit) [30]. The relative abundance of each virus was quantified as RPM, calculated using the formula: "(total virus reads / total reads) × 1 million". To determine the coverage and sequencing depth of each virus, the sequencing reads were aligned to the respective reference virus genome sourced from NCBI using BWA MEM v0.7.17 (http://bio-bwa.sourceforge.net) [31]. Subsequently, SAMtools v1.7 (https://github.com/samtools/samtools) was utilized to calculate essential metrics, including the percentage of mapped reads and the coverage depth [32].

Oxford Nanopore Sequencing and QitanTech Nanopore Sequencing Data Analysis: The raw FAST5 files generated by sequencing underwent basecalling with Guppy v2.2.7 (Oxford Nanopore Technologies) to convert into FASTQ files. NanoPlot v1.32.1 (https://github.com/wdecoster/NanoPlot) was utilized to assess the sequencing reads, including their counts, length, and quality. To facilitate comparison across different sequencing methodologies, the sequencing data were normalized to ensure an equivalent number of sequenced bases using SeqKit v2.4.0 (https://github.com/shenwei356/seqkit) [27]. The primers of the SMART-RNA-Metavirome were trimmed using Cutadapt v1.18 (https://cutadapt.readthedocs.io/) as described above [28]. The reads were compared against the nr databases sourced from NCBI, using Diamond blastx v0.9.21 (https://github.com/bbuchfink/diamond) with a cut-off E-value of 1 × 10^–5^ [29]. The relative abundance of the viruses was calculated as RPM as described above. To determine the coverage and sequencing depth of each virus, Minimap2 v2.22 (https://github.com/lh3/minimap2)was employed to align the reads to reference virus genomes obtained from NCBI [33]. SAMtools v1.7 (https://github.com/samtools/samtools) was then used to calculate the percentage of mapped reads and the coverage depth [32].

### Detection of DENV-infected *Ae. albopictus*

To evaluate the detection sensitivity of the SMART-RNA-Metavirome platform, we employed a stringent testing model involving pools each containing a single DENV-infected *Ae. albopictus* mosquito. Specifically, pools of DENV-infected *Ae. albopictus* were rinsed with PBS solution (Cat. No. C10010500BT, Gibco, Grand Island, NY, USA) prior to homogenization using a tissue grinder in PBS solution. In the following, total RNA was extracted using AG RNAex Pro Reagent (Cat. No. AG21102, Accurate Biology, Hunan, China), following the manufacturer's instructions. The quantity and quality of the extracted RNA were assessed using a NanoDrop spectrophotometer. For RT-qPCR, RNA (1 μg) was used to quantify the DENV-1 titers. The detection capabilities reported by the SMART-RNA-Metavirome platform for DENV-infected mosquitoes samples are based on these pools with diverse RT-qPCR Ct-values. Specifically, RNA (1.8 μg) of these pools with diverse RT-qPCR Ct-values was utilized to generate sequencing libraries using the SMART-RNA-Metavirome protocol. These libraries were then sequenced using Oxford Nanopore sequencing technology, as described above.

### DENV reads mapping

Nanopore sequencing data analysis was conducted as described above. To determine the percentage of DENV reads in each sample, Minimap2 v2.22 (https://github.com/lh3/minimap2) was used to align the reads to the reference virus genome of NV46 isolate [33]. SAMtools v1.7 (https://github.com/samtools/samtools) was then employed to determine the number of mapped reads and the coverage depth [32]. Consensus sequences were subsequently generated using BCFtools v1.9 (https://github.com/samtools/bcftools) and further polished by Pilon v1.24 (https://github.com/broadinstitute/pilon/) [34].

To determine the optimal sequencing yield necessary for generating high-quality and reliable consensus viral genomes, the sequencing data from the sample with an RT-qPCR Ct-value of 21.6 was randomly down-sampled to specific percentages or numbers of sequencing reads. For each down-sampling dataset, SeqKit v2.4.0 (https://github.com/shenwei356/seqkit) was used to calculate the number of reads[27]. Thereafter, Minimap2 v2.22 (https://github.com/lh3/minimap2) was applied to align the reads to the reference virus genome of NV46 isolate[33]. SAMtools v1.7 (https://github.com/samtools/samtools) was then used to determine the average DENV depth at each position across the genome and the percentage of genome coverage at 20 × [32]. Consensus sequences were generated using BCFtools v1.9 (https://github.com/samtools/bcftools) and further polished with Pilon v1.24 (https://github.com/broadinstitute/pilon/) [32, 34]. After refinement, the consensus sequences were blast against the reference virus genome of NV46 isolate to determine their identity.

### SMART-RNA-Metavirome platform for clinical serum samples detection

Serum samples were collected from patients diagnosed with dengue fever during outbreaks in Guangdong Province between September and November 2019. Prior to storage at -80℃, all specimens were confirmed to be positive for DENV using NS1 antigen detection kits and for the presence of anti-DENV IgM or IgG antibodies through standard diagnostic protocols. Subsequently, each serum sample (160 µl) was used to extract total RNA using the AG RNAex Pro Reagent (Cat. No. AG21102, Accurate Biology, Hunan, China) according to the manufacturer’s instructions. Then, the extracted total RNA (90 ng) was utilized for quantification of viral titers through RT-qPCR. Additionally, another 90 ng of total RNA was employed to construct sequencing libraries using SMART-RNA-Metavirome. These libraries were then sequenced using Oxford Nanopore technology and analyzed as described above.

### SMART-RNA-Metavirome platform for viral isolates detection

The JEV strain (SA14-14-2, GenBank accession No. AF315119.1) was stored within our laboratory. The ZIKV strain (GenBank accession No. KU820899.2) provided by the Center for Disease Control and Prevention of Guangdong Province, was originally isolated from a patient in China in February 2016 and classified as the Asian lineage. The JEV was propagated in Vero cells maintained at 37℃ with 5% CO_2_ in DMEM medium (Cat. No. C11995500BT, Thermo Fisher Scientific, Waltham, MA, USA) supplemented with 2% fetal bovine serum (FBS) (Cat. No. FSP500, ExCell Bio, Suzhou, China). Meanwhile, the ZIKV was cultivated in C6/36 cells maintained at 28℃ in RPMI 1640 medium (Cat. No. C11875500BT, Thermo Fisher Scientific, Waltham, MA, USA) supplemented with 2% FBS. After harvesting the viral isolates, total RNA was extracted using AG RNAex Pro Reagent (Cat. No. AG21102, Accurate Biology, Hunan, China) according to the manufacturer’s instructions. Subsequently, the total RNA of JEV viral isolates was diluted with total RNA extracted from uninfected Vero cells to achieve a range of concentrations: 10^8.4^, 10^7.7^, 10^6.4^, 10^5.3^, 10^4.3^, and 10^4.1^ copies/µl, as determined by RT-qPCR. Similarly, the total RNA of ZIKV viral isolates was diluted with total RNA from uninfected C6/36 cells to concentrations of 10^6.6^, 10^5.5^, 10^4.6^, 10^3.6^, 10^2.6^, 10^1.7^, and 10^0.6^ copies/µl, as determined by RT-qPCR. For sequencing, RNA (2 μg) from diluted JEV viral isolates and RNA (620 ng) from diluted ZIKV viral isolates were used to construct sequencing libraries using SMART-RNA-Metavirome. These libraries were then sequenced using Oxford Nanopore technology and analyzed as described above.

## Results

### SMART-RNA-Metavirome platform: compatible with NGS and TGS

**Fig. 1:**
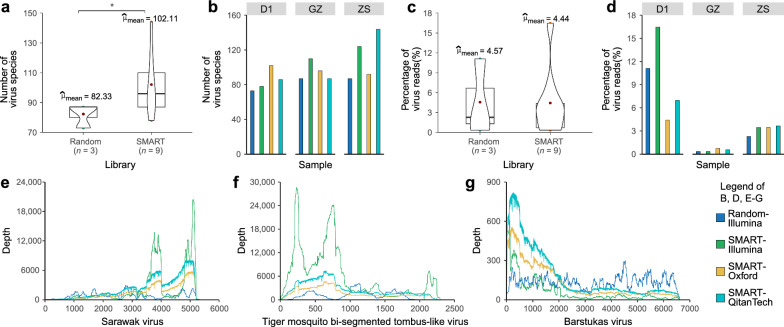
SMART-RNA-Metavirome effectively enhances the compatibility of sequencing platforms (including Illumina, Oxford Nanopore, QitanTech Nanopore) for detecting the metavirome in laboratory models of *Ae. albopictus* infected DENV (D1), and the wild populations of *Ae. albopictus* sampled in Guangzhou (GZ) and Zhongshan (ZS). **a–d** Comparison of the number of virus species and the percentage of viral reads obtained from the SMART-RNA-Metavirome library combined with Illumina, Oxford Nanopore, and QitanTech Nanopore as well as the random primer library combined with Illumina sequencing platforms. **e–g** Depth plots of Sarawak virus, Tiger mosquito bi-segmented tombus-like virus, and Barstukas virus generated by SMART-RNA-Metavirome library combined with Illumina, Oxford Nanopore, and QitanTech Nanopore as well as the random primer library combined with Illumina sequencing platforms. Random—Random primer library; SMART—SMART-RNA-Metavirome library; Random-Illumina—Random primer library combined with Illumina sequencing; SMART-Illumina—SMART-RNA-Metavirome library combined with Illumina sequencing; SMART-Oxford—SMART-RNA-Metavirome library combined with Oxford Nanopore sequencing; SMART-QitanTech—SMART-RNA-Metavirome library combined with QitanTech Nanopore sequencing

The SMART-RNA-Metavirome system is a practical RNA metavirome technology devised to enrich the composition and diversity of RNA viromes extracted from their respective host metatranscriptomes. To systematically evaluate the transcriptional fidelity and amplification efficiency of this platform in comparison to conventional random primer-based approaches during cDNA library preparation, we implemented four distinct sequencing workflows across three sample categories: (1) laboratory models of *Ae. albopictus* infected with DENV (D1), (2) field-collected *Ae. albopictus* populations from Guangzhou (GZ), and (3) Zhongshan (ZS). These workflows comprised: the random primer-based method followed by Illumina sequencing (Random-Illumina); the SMART-RNA-Metavirome-based method coupled with Illumina sequencing (SMART-Illumina); the SMART-RNA-Metavirome-based Oxford Nanopore sequencing (SMART-Oxford); and the SMART-RNA-Metavirome-based QitanTech Nanopore sequencing (SMART-QitanTech). For the three samples, the random primer-based Illumina sequencing generated raw read counts of 35,468,726, 35,992,483, and 34,359,495, respectively. In contrast, the SMART-RNA-Metavirome-based Illumina sequencing produced 32,794,522, 26,612,372, and 9,897,374 raw reads (Additional file 1). The SMART-RNA-Metavirome-based Oxford Nanopore sequencing yielded 576,650, 662,937, and 822,070 raw reads, with average read lengths of 967 bp, 1047 bp, and 926 bp, read lengths N50 of 1209 bp, 1376 bp, and 1160 bp, and maximum read lengths of 201,697 bp, 22,202 bp, and 26,561 bp, respectively. The SMART-RNA-Metavirome-based QitanTech Nanopore sequencing generated 1,853,283, 2,306,930, and 2,118,523 raw reads, with average read lengths of 766 bp, 760 bp, and 717 bp, read lengths N50 of 884 bp, 875 bp, and 820 bp, and maximum read lengths of 12,839 bp, 9706 bp, and 10,748 bp, respectively (Additional file 1). Given the substantial disparities in sequencing data volume and read length across the methods, we normalized the total sequenced bases through subsampling to ensure comparability. Subsequent metavirome analysis revealed a statistically significant increase in viral species diversity for SMART-RNA-Metavirome compared to the random primer-based method (Welch’s *t*-test, *P = 0.04; Fig. 1a). Specially, for the D1 sample, the random primer-based method detected 73 viral species, while SMART-RNA-Metavirome identified 78, 102, and 86 species across the three sequencing platform. For the GZ sample, the random primer-based method detected 87 species, in contrast to 110, 96, and 87 species with SMART-RNA-Metavirome. For the ZS sample, the random primer-based method detected 87 species, whereas SMART-RNA-Metavirome identified 124, 92, and 144 species (Fig. 1b, Additional file 1). Although the proportion of viral sequences identified by both methods was not statistically comparable (Welch’s *t*-test, *P* = 0.97; Fig. 1c), numerical trends indicated higher proportions in the GZ and ZS samples when using SMART-RNA-Metavirome (Fig. 1d). Notably, SMART-RNA-Metavirome provided sufficient coverage and depth to assemble complete viral genomes with high accuracy. For instance, a marked increase in sequencing depth was observed for Sarawak virus, Tiger mosquito bi-segmented tombus-like virus, and Barstukas virus in the SMART-RNA-Metavirome sequencing data (Fig. 1e–g).

**Fig. 2:**
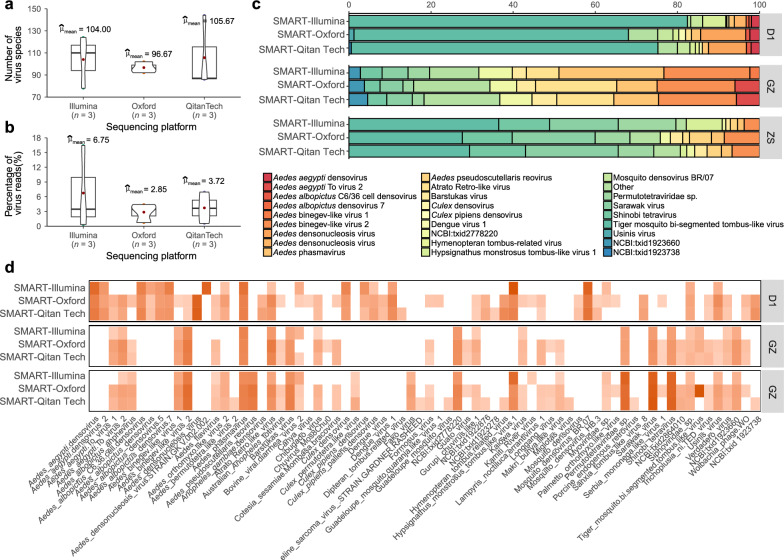
SMART-RNA-Metavirome is compatible with TGS sequencing platforms (including Oxford Nanopore and QitanTech Nanopore sequencing) for detecting the metavirome in laboratory models of *Ae. albopictus* infected with DENV (D1), and the wild populations of *Ae. albopictus* sampled in Guangzhou (GZ) and Zhongshan (ZS). **a, b** Comparison of the number of virus species and the percentage of viral reads obtained from the SMART-RNA-Metavirome library combined with Illumina, Oxford Nanopore, and QitanTech Nanopore sequencing. **c** Proportion of virus species detected in D1, GZ and ZS *Ae. albopictus*. Display only the top 10 most abundant viruses identified in each sample, and all viruses that do not fall within this top 10 list are collectively grouped and labeled as "Others". The complete information is in Additional file 2. **d** The heatmap displays the viral RPMs (RPMs ≥ 10 were shown). SMART-Illumina—SMART-RNA-Metavirome library combined with Illumina sequencing; SMART-Oxford—SMART-RNA-Metavirome library combined with Oxford Nanopore sequencing; SMART-QitanTech—SMART-RNA-Metavirome library combined with QitanTech Nanopore sequencing

To evaluate the compatibility of SMART-RNA-Metavirome with TGS sequencing platforms, we conducted a comprehensive comparison of its performance across Illumina, Oxford Nanopore, and QitanTech Nanopore technologies. Despite differences in total read counts and read lengths, viral species diversity and sequence proportions remained comparable across different platforms (Welch’s *F*-test, *P* = 0.61 and 0.68 respectively; Fig. 2a and b). The composition of viral species was highly consistent: for instance, the Aedes binegev-like virus 2 constituted approximately 20.9%, 19.0%, and 19.0% of the viral reads in the GZ population across all three sequencing platforms, respectively. Similarly, Shinobi tetravirus accounted for roughly 12.4%, 12.1%, and 13.6% of the viral reads in the GZ population (Fig. 2c, Additional file 2). Additionally, the viral number of reads mapped per million input reads (RPM) values for viral species generated by these platforms using the SMART-RNA-Metavirome-based approach exhibited consistent patterns (Fig. 2d, Additional file 2). These results confirm that SMART-RNA-Metavirome integrates efficiently with TGS technologies, facilitating robust and versatile virome characterization.

### SMART-RNA-Metavirome platform for DENV-infected *Ae. albopictus* detection

**Fig. 3:**
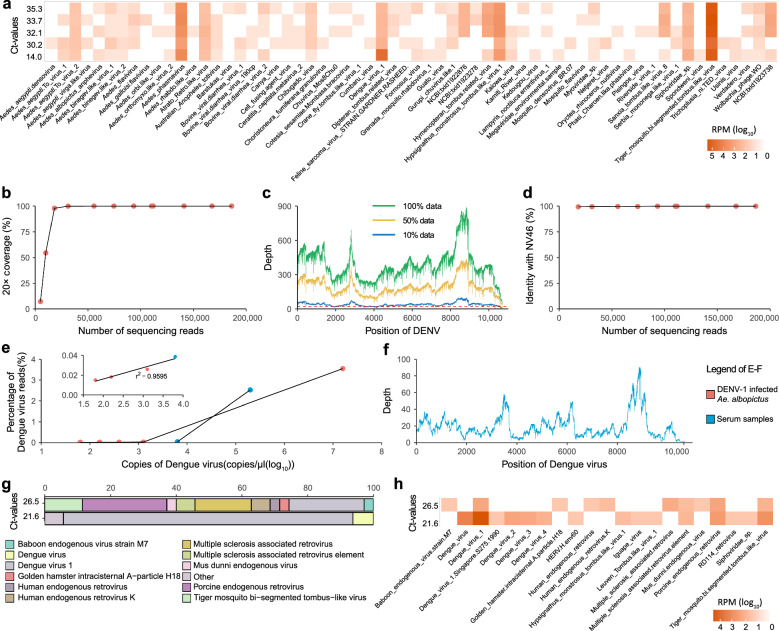
Utilization of SMART-RNA-Metavirome platform in detecting DENV genomes from infected *Ae. albopictus* and clinical serum samples. **a** The heatmap shows the RPMs of viruses detected in infected *Ae. albopictus* with various DENV-1 titers. **b–d** Analytical sensitivity of the SMART-RNA-Metavirome platform integrated with Oxford Nanopore sequencing for detection of the DENV-1 genome in infected *Ae. albopictus* with an RT-qPCR Ct-value of 14 after randomly down-sampling to a specific percentage or number of sequencing reads (**b** Percentage of genome coverage at 20 × depth with increasing number of sequencing reads after sampling. **c** Average depth of DENV at each mapped read position across the genome after sampling. **d** Identity of the DENV-1 consensus genome with the reference N46V genome after sampling.) **e** Correlation between viral titers and percentages of the viral reads within sequencing data of DENV-infected *Ae. albopictus* and serum samples. **f** DENV genome average depth at each position of the mapped reads detected from the serum sample with a Ct-value of 21.6. **g** Proportion of virus species detected in serum samples. Display only the top 10 most abundant viruses identified in each sample, and all viruses that do not fall within this top 10 list are collectively grouped and labeled as "Others". The complete information is in Additional file 7. **h** The heatmap shows the RPMs of virus detected in serum samples

The infected mosquito samples with different DENV-1 titers exhibited the presence of DENV viral sequences and various other virus sequences, as depicted in Fig. 3a and Additional file 3. Among the detected viral sequences, Tiger mosquito bi-segmented tombus-like virus accounted for the highest proportion (Additional file 4). For samples with a Ct-value exceeding 30.0, this specific virus sequence constituted over 90% of all detected sequences, with DENV sequences contributing to less than 1%. However, for samples exhibiting a Ct-value of 14.0, the proportion of Tiger mosquito bi-segmented tombus-like virus sequences decreased to 72.2%, while the proportion of DENV sequences increased to 23.3% (Additional file 4).

Furthermore, to determine the optimal sequencing yield required for generating high-quality and reliable consensus viral genomes, we conducted a comprehensive analysis of the data generated by the SMART-RNA-Metavirome platform, specifically targeting samples of *Ae. albopictus* infected with DENV-1 that had been pre-identified with an RT-qPCR Ct-value of 14.0. Notably, when the sequencing reads reached 18,100, representing approximately 10% of the total sequencing data, the viral genome coverage at 20 × depth abruptly plateaued. This allowed for the recovery of nearly the full-length (97.84%) viral genome (Fig. 3b and c). This high recovery enabled us to reconstruct the complete DENV genome with an identity of 99.23% compared to the reference DENV-1 genome of the N46V strain, which had been previously assembled based on Illumina sequencing in our earlier study (Fig. 3d). This result underscores the remarkable capability of the SMART-RNA-Metavirome platform in generating precise and comprehensive viral genomes, even from the samples with limited viral loads.

Notably, in mosquito samples exhibiting low DENV-1 titers, a robust correlation was observed between the viral loads of DENV-1 and the percentage of viral reads (*r*^2^ = 0.9991, linear regression analysis) (Fig. 3e and Additional file 5). This significant correlation demonstrates the high sensitivity of the SMART-RNA-Metavirome platform in detecting even minute quantities of viral RNA. Even in a sample with a Ct-value of 35.3, an impressive 99.88% genome coverage was achieved, with average and maximum depths of 3.9 × and 14 × , respectively. Such a high level of coverage is sufficient for accurate viral genome identification, thereby further highlighting the exceptional sensitivity of the SMART-RNA-Metavirome platform in detecting viral genomes from mosquito samples.

### SMART-RNA-Metavirome platform for clinical serum samples detection

After successfully validating the SMART-RNA-Metavirome platform using the aforementioned infected mosquito model, we applied it to clinical serum samples. Specifically, we selected serum samples from dengue patients with RT-qPCR Ct-values of 21.6 and 26.5 for the SMART-RNA-Metavirome analysis. By comparing the sequencing results from these serum samples with those obtained from DENV-infected *Ae. albopictus*, we observed a comparable trend: an increase in the proportion of viral reads correlated with higher viral titers (Fig. 3e and Additional file 5 and 6). Furthermore, our data revealed a robust correlation (*r*^2^ = 0.9595) between the proportion of viral reads and viral titers within a specific range, based on both serum samples and DENV-infected *Ae. albopictus* (Fig. 3e and Additional files 5 and 6).

Moreover, a comprehensive analysis of the serum sample with an RT-qPCR Ct-value of 21.6 revealed that 867 reads could be mapped back to the DENV reference genome (N46V, GenBank Accession No. KX458014.1) using a reference-based approach (Fig. 3f). Upon performing a Diamond blast analysis against the nonredundant protein (nr) database, a total of 1,288 reads were identified as belonging to DENV. Notably, 1,184 reads were accurately classified as DENV-1, while an additional 20 reads matched serotypes DENV-2, DENV-3, or DENV-4. Additionally, 84 reads were identified as DENV without specific serotype information (Fig. 3g, h and Additional file 7). These results underscore the robustness of Nanopore sequencing, which, despite its inherent error rate, is not only capable of virus species identification but also serotype determination, independent of reference genomes. This finding highlights the powerful capabilities of SMART-RNA-Metavirome integrated with Oxford Nanopore sequencing in identifying the viral pathogens responsible for emerging infections.

### SMART-RNA-Metavirome platform for viral isolates detection

**Fig. 4:**
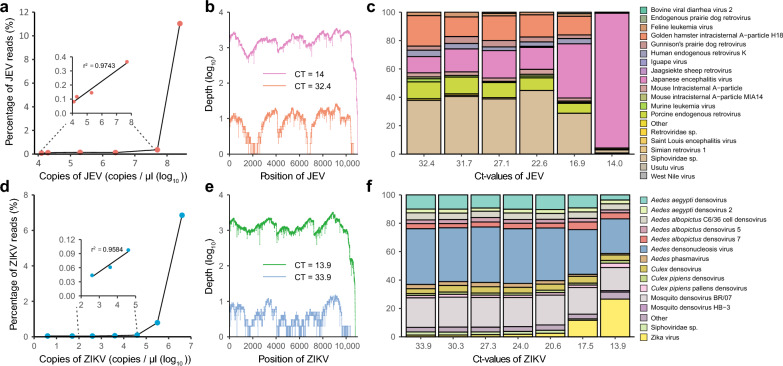
The utilization of SMART-RNA-Metavirome platform for detecting viral genomes of different species. **a** Correlation between viral titers and percentages of the viral reads within sequencing data of the JEV-diluted solutions with different titers. **b** Genome average JEV depth at each position of the mapped reads for the samples with Ct-values of 14.0 and 32.4. **c** Proportion of virus species detected in JEV samples. Display only the top 10 most abundant viruses identified in each sample, and all viruses that do not fall within this top 10 list are collectively grouped and labeled as "Others". The complete information is in Additional file 10. **d** Correlation between viral titers and percentages of the viral reads within sequencing data of ZIKV-diluted solutions with different titers. **e** Genome average ZIKV depth at each position of the mapped reads for the samples with Ct-values of 13.9 and 33.9. **f** Proportion of the virus species detected in ZIKV samples. Display only the top 10 most abundant viruses identified in each sample, and all viruses that do not fall within this top 10 list are collectively grouped and labeled as "Others". The complete information is in Additional file 11.

When subjected to the diluted viral solutions, a lesser extent was more obviously observed than anticipated based on the dilution ratios although both JEV and ZIKV sequences exhibited reduced proportions in the sequencing results. For instance, upon diluting JEV to 1/100, the proportion of JEV reads decreased from 11.03% to 0.13%, representing a reduction to only 1/84.8 of the original proportion (Fig. 4a and Additional file 8). Similarly, following a 1/10 dilution of ZIKV, the proportion of ZIKV reads declined from 6.85% to 0.80%, indicating only a 1/8.56 reduction relative to the 1/10 dilution rate (Fig. 4d and Additional file 9). In other words, even when dealing with samples containing low viral titers, SMART-RNA-Metavirome exhibited remarkable efficiency in reverse transcription and enrichment for capturing viral sequences. Notably, a strong correlation existed between viral titers and the percentages of viral reads within sequencing data for samples characterized by low virus titers (*r*^2^ = 0.9743 and 0.9584, respectively, for JEV and ZIKV, as determined by linear regression analysis) (Fig. 4a and d, Additional files 8 and 9). For the samples with high virus titers, the SMART-RNA-Metavirome approach achieved full genome coverage of viruses at a high level of depth (Fig. 4b and e). In the case of the JEV viral diluted solution with an RT-qPCR Ct-value of 14, the proportion of JEV reads accounted for 11.03% of the total reads and 94.97% of the viral reads, enabling the construction of the full JEV genome with an impressive identity match of 99.74% to the reference genome (Fig. 4b and 3c, Additional file 10). Similarly, for ZIKV viral diluted solution with an RT-qPCR Ct-value of 13.9, the proportion of ZIKV reads amounted to 6.85% of the total reads and 26.58% of the viral reads, resulting in the assembly of the full ZIKV genome that shared an identity match as high as 99.64% with the reference genome (Fig. 4e and f, Additional file 11). Apart from JEV and ZIKV viral reads, the diluted JEV and ZIKV solutions with different titers exhibited the presence of various other virus sequences, as depicted in Fig. 4c and f, and Additional file 12.

## Discussion

Metavirome appears as a cornerstone technology for identifying and predicting unknown pathogens, as well as for the early detection of the emergence and re-emergence viral infectious diseases [35]. The RNA metavirome focuses on non-targeted sequencing, where the generation of a cDNA library from RNA constitutes an essential step [36]. Preliminary, SMART-9n has proven its efficacy in detecting various known viral isolates, including ZIKV, YFV, and SARS-CoV-2 [23]. In this study, we developed and systematically evaluated a practical non-targeted SMART-RNA-Metavirome platform compatible with NGS and TGS. We employed SMART-9n for the library preparation of RNA metavirome, offering a novel method to compared to traditional library construction methodologies. Our findings indicated that the SMART-RNA-Metavirome approach exhibited a superior capacity to capture a greater diversity of RNA viromes and demonstrated commendable compatibility with NGS and TGS technologies. Notably, TGS technologies possess the proficiency to generate long reads. Consequently, when integrated into the SMART-RNA-Metavirome approach, they facilitates comprehensive coverage and enables accurate reconstruction of complete viral genomes. Moreover, it even exhibits superior sequencing depth when compared to NGS Illumina sequencing commonly employed in metavirome analysis. Therefore, the SMART-RNA-Metavirome platform demonstrates enhanced capabilities in preparing high-quality data for the discovery of RNA viruses. Notably, a cutting-edge AI-based metagenomic mining technique has been devised and validated to substantially augment the exploration of diversity within the global RNA virosphere, thereby streamlining data excavation and analysis [37]. Looking ahead, the future holds promise for the synergistic application of the SMART-RNA-Metavirome platform for data preparation, coupled with the AI-powered LucaProt system for data mining, to further advance our understanding of RNA virosphere diversity.

In recent decades, the dramatic emergence and re-emergence of severe infectious diseases, partly attributed to arboviruses such as DENV, ZIKV, and CHIKV, pose significant threats to global health [38–40]. These pathogens, which have been defined as Priority Pathogens by the World Health Organization (WHO), act as pre-warning pathogen X due to their potential impact [40]. However, the atypical clinical manifestations of these pathogen-induced infections have hampered early virus detection and subsequently the effective management of diseases and outbreaks [39]. Moreover, the inherent error-prone characteristics of RNA-dependent RNA polymerases (RdRPs) in RNA viruses, coupled with the notably low viral titers present in vector mosquitoes, have posed substantial obstacles to the effective surveillance and monitoring of arboviruses within these insect vectors. Although Nanopore sequencing has shown remarkable success in the targeted detection of arboviruses, such as ZIKV [15], DENV-2 [16], CHIKV [17], and YFV [18], it currently relies on prior knowledge of specific viruses and has primarily shown efficacy only in clinical specimens or viral isolates cultivated in cellular systems. Therefore, it is imperative to develop an innovative technological platform for detecting both known and unknown arboviruses in wild mosquito populations with low viral titers through a non-targeted metavirome sequencing approach. In our investigation, the application of SMART-RNA-Metavirome integrated with Oxford Nanopore sequencing to *Ae. albopictus* infected with DENV-1 yielded sufficient coverage for accurate viral genome identification, even in a sample exhibiting a Ct-value of 35.3. This result indicates the exceptional capability of the SMART-RNA-Metavirome platform for detecting arboviruses in wild mosquito specimens. However, the reported detection capability is a preliminary estimate, and subsequent studies will establish a formal limit of detection (LOD) based on technical replicates. Furthermore, the utilization of SMART-RNA-Metavirome platform in clinical serum samples not only enabled the identification of virus species but also facilitated the further determination of serotypes. The discrimination of serotypes among mosquito-borne viruses is important for predicting disease severity and guiding clinical management, owing to significant differences in pathogenicity across serotypes. For instance, infection with DENV-2 is associated with a higher incidence of severe outcomes such as dengue hemorrhagic fever (DHF), and heterotypic reinfection with a different serotype substantially increases the risk of severe morbidity and mortality. Furthermore, surveillance and tracking of emerging infectious diseases or potential pandemics are particularly crucial for the early identification of public health threats. Compared with traditional pathogen detection methods, this platform can reveal the non-targeted full virus spectrum, even specific viral serotypes, which is helpful for virus phylogenetic source tracing and risk assessment, and shows the potential of early warning of new strain outbreaks. From an epidemiological perspective, this platform facilitates a paradigm shift from reactive patient-based detection to proactive early outbreak alerting, thereby transforming passive disease surveillance into an active monitoring system with preemptive intervention capacity.

Nevertheless, the SMART-RNA-Metavirome platform exhibits certain limitations. While SMART-RNA-Metavirome prevents rRNA amplification through its full-length cDNA capture design, fragmented sequences lacking the 5' cap structure—resulting from sample processing or handling—cannot undergo reverse transcription or amplification. This limitation consequently diminishes viral sequence recovery in downstream analyses. On the other hand, although SMART-RNA-Metavirome employs non-targeted sequencing, the inevitable sequence amplification bias introduced by PCR may affect the accuracy of relative abundance estimates among pathogens in subsequent sequencing analyses. While the observed decline in viral sequence representation is less pronounced than the theoretical dilution factor, which facilitates the detection of low-abundance viral sequences, this amplification bias may also introduce potential errors in estimating the relative abundances of the detected viral species under low-titer conditions. In addition, despite the platform's relatively high sensitivity, viral sequences consistently constitute a minor fraction of individual sample libraries, whereas host-derived background sequences dominate. This bias was observed across all sample types tested in this study, including DENV-infected *Ae. albopictus*, viral isolates, and clinical serum samples. Specially, in low viral titer samples, viral reads may account for < 1% of total sequencing output. To mitigate this issue, in our future studies, host background depletion strategies will be implemented to enhance the viral signal recovery. Regarding clinical samples, our platform has undergone a preliminary application for pathogen detection in serum, nevertheless, clinical samples encompass a diverse array of types, including saliva, urine, and cerebrospinal fluid. Therefore, to assess the generalizability of the SMART-RNA-Metavirome platform across different categories of human samples, it is essential to incorporate additional clinical specimens for further optimization of its performance.

## Conclusions

The SMART-RNA-Metavirome platform established in this study offers several advantages, including superior capture of a greater diversity of RNA viromes, high compatibility with both NGS and TGS technologies for comprehensive coverage and accurate viral genome reconstruction, enhanced sequencing depth compared to conventional methods, and proficiency in detecting both known and unknown arboviruses, even in samples with low viral titers such as those from wild mosquitoes and clinical sera. Leveraging these strengths, this platform facilitates the monitoring, risk assessment, and early warning of potential inter-species and/or cross-regional transmissions of RNA viruses, it can even provide pre-warning for pathogen X and other unknown emerging viruses, from mosquito vectors and clinical samples. Furthermore, it enhances the elucidation of geographical ecological patterns and the underlying drivers of RNA virome diversity.

## Supplementary Information

**Additional file1: Table S1.** Sequencing outcomes utilizing SMART-RNA-Metavirome in combination with Illumina, Oxford Nanopore, and QitanTech Nanopore technologies, alongside a random primer library coupled with Illumina sequencing technology for the detection of wild *Ae. albopictus* populations. ^a) ^The number of raw sequencing reads; ^b) ^The mean length of raw sequencing reads; ^c) ^The N50 length of raw sequencing reads; ^d) ^The maximal length observed among raw sequencing reads; ^e) ^The number of sequencing reads after normalization and sampling; ^f) ^The number of viral reads within the sampled reads; ^g) ^The proportion of viral reads as a percentage; ^h) ^The total number of distinct virus species identified; ^i-k) ^Refer to DENV-infected *Ae. albopictus*, and wild populations of *Ae. albopictus* collected from Guangzhou (GZ) and Zhongshan (ZS); ^l) ^Random primer library combined with Illumina sequencing; ^m) ^SMART-RNA-Metavirome library combined with Illumina sequencing; ^n) ^SMART-RNA-Metavirome library combined with Oxford Nanopore sequencing; ^o) ^SMART-RNA-Metavirome library combined with QitanTech Nanopore sequencing.

**Additional file2: Table S2.** Detection of RNA viromes in DENV-infected *Ae. albopictus* and wild populations of *Ae. albopictus* using SMART-RNA-Metavirome coupled with Illumina, Oxford Nanopore, and QitanTech Nanopore technologies, as well as a random primer library combined with Illumina Sequencing technology: Virus species identification, RPM, and Proportion of virus detected. ^a-c) ^Refer to DENV-infected *Ae. albopictus*, and wild populations of *Ae. albopictus* collected from Guangzhou (GZ) and Zhongshan (ZS); ^d) ^Random primer library combined with Illumina sequencing; ^e) ^SMART-RNA-Metavirome library combined with Illumina sequencing; ^f) ^SMART-RNA-Metavirome library combined with Oxford Nanopore sequencing; ^g) ^SMART-RNA-Metavirome library combined with QitanTech Nanopore sequencing

**Additional file3: Table S3.** Detection of RNA virome in DENV-infected *Ae. albopictus* using SMART-RNA-Metavirome platform integrated with Oxford Nanopore sequencing: Virus species identification, RPM, and Proportion of virus detected

**Additional file4: Fig S1.** Proportion of virus species identified in infected *Ae. albopictus* using the SMART-RNA-Metavirome platform compatible with Oxford Nanopore sequencing technology

**Additional file5: Table S4.** RT-qPCR and sequencing outcomes of DENV detection in infected *Ae. albopictus* using SMART-RNA-Metavirome platform integrated with Oxford Nanopore sequencing. ^a) ^The Ct-values of DENV detected by RT-qPCR; ^b) ^Log10-transformed copies of DENV; ^c) ^Number of sampling sequencing reads; ^d)^Average length of raw sequencing reads; ^e) ^Maximum length of raw sequencing reads; ^f) ^Number of DENV-1 reads within the sampled reads; ^g) ^Percentage of DENV-1 reads in the sampled reads

**Additional file6: Table S5.** RT-qPCR and sequencing outcomes of DENV detection in clinical serum samples using SMART-RNA-Metavirome platform integrated with Oxford Nanopore sequencing. ^a)^The Ct-values of DENV detected by RT-qPCR; ^b)^Log10-transformed copies of DENV; ^c)^Number of sampling sequencing reads; ^d)^Average length of raw sequencing reads; ^e)^Maximum length of raw sequencing reads; ^f)^Number of DENV-1 reads within the sampled reads; ^g)^Percentage of DENV-1 reads in the sampled reads

**Additional file7: Table S6.** Detection of RNA virome in clinical serum samples using SMART-RNA-Metavirome platform integrated with Oxford Nanopore sequencing: Virus species identification, RPM, and Proportion of virus detected

**Additional file8: Table S7.** RT-qPCR and sequencing outcomes of JEV detection in viral isolate samples with different titers using SMART-RNA-Metavirome platform integrated with Oxford Nanopore sequencing. ^a)^The Ct-values of JEV detected by RT-qPCR. ^b)^Log10-transformed copies of JEV; ^c)^Number of raw sequencing reads; ^d)^Average length of raw sequencing reads; ^e)^Maximum length of raw sequencing reads; ^f)^Number of JEV reads within the raw sequencing reads; ^g)^Percentage of JEV reads in raw sequencing reads

**Additional file9: Table S8.** RT-qPCR and sequencing outcomes of ZIKV detection in viral isolate samples with different titers using SMART-RNA-Metavirome platform integrated with Oxford Nanopore sequencing. ^a)^The Ct-values of ZIKV detected by RT-qPCR; ^b)^Log10-transformed copies of ZIKV; ^c)^Number of raw sequencing reads; ^d)^Average length of raw sequencing reads; ^e)^Maximum length of raw sequencing reads; ^f)^Number of ZIKV reads within the raw sequencing reads; ^g)^Percentage of ZIKV reads in raw sequencing reads

**Additional file10: Table S9.** Detection of RNA virome in JEV viral isolates with different titers using SMART-RNA-Metavirome platform integrated with Oxford Nanopore sequencing: Virus species identification, RPM, and Proportion of virus detected

**Additional file11: Table S10.** Detection of RNA virome in ZIKV viral isolates with different titers using SMART-RNA-Metavirome platform integrated with Oxford Nanopore sequencing: Virus species identification, RPM, and Proportion of virus detected

**Additional file12: Fig S2.** Application of the SMART-RNA-Metavirome platform for detecting viral genomes across various species. **A** Heatmap representing the RPM of virus detected in JEV isolate with different titers. **B** Heatmap displaying the RPM of virus detected in ZIKV isolate with different titers.

## Data Availability

The datasets generated during the current study are available in the National Microbiology Data Center (NMDC) at https://nmdc.cn/, reference number NMDC10019851, NMDC10019495, NMDC10019496, NMDC10019497, NMDC10019498 and NMDC10019499.

## Abbreviations

CHIKV: Chikungunya virus
coxI: Cytochrome c oxidase subunit I mitochondrial gene
DENV: Dengue virus
dNTP: Deoxyribonucleotide triphosphates
EBOV: Ebola virus
FBS: Fetal bovine serum
GZ: Guangzhou
JEV: Japanese encephalitis virus
LNA: Locked nucleic acid
LOD: Limit of detection
NCBI: National Center for Biotechnology Information
NGS: Next-generation sequencing
nr: Nonredundant protein
PBS: Phosphate buffered saline
RCA: Rolling circle amplification
RdRP: RNA-dependent RNA polymeras
RPM: Number of reads mapped per million input reads
rRNA: Ribosomal RNA
RT-PCR: Reverse transcription-polymerase chain reaction
RT-qPCR: Reverse transcription quantitative polymerase chain reaction
SARS-CoV-2: Severe acute respiratory syndrome coronavirus 2
SISPA: Sequence-independent single primer amplification
SMART: Switching mechanism at the 5′ end of the RNA transcript
TGS: Third-generation sequencing
WHO: World Health Organization
YFV: Yellow fever virus
ZIKV: Zika virus
