# Supplementary figures and images for: SMART-RNA-Metavirome: a practical RNA metavirome platform compatible with high-throughput sequencing of both short and long reads
Source: Infect Dis Poverty. 2025 Oct 10;14:101. doi: 10.1186/s40249-025-01371-z (PMC12512776; doi:10.1186/s40249-025-01371-z)

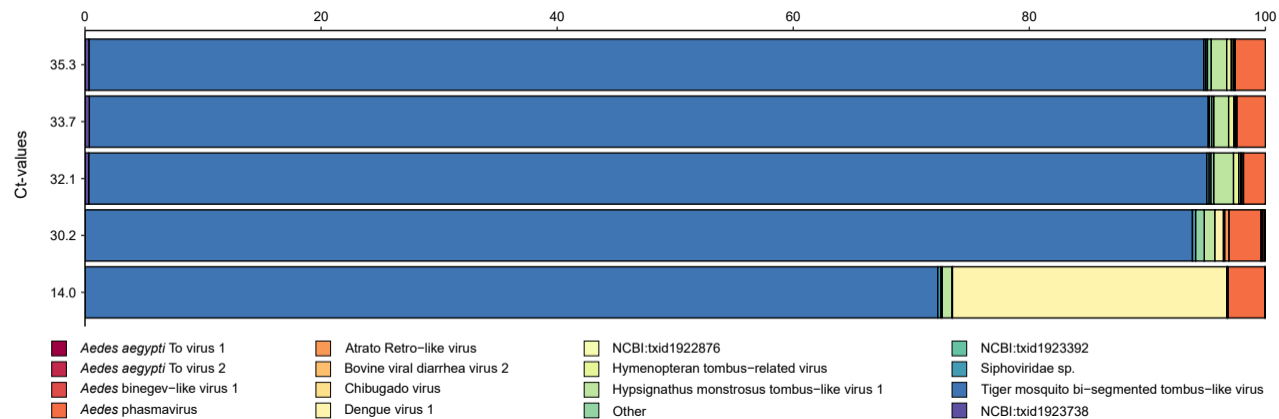

Supplement: Supplementary file 4 — Additional file4: Fig S1. Proportion of virus species identified in infected Ae. albopictus using the SMART-RNA-Metavirome platform compatible with Oxford Nanopore sequencing technology [file 40249_2025_1371_MOESM4_ESM.pdf]

# B

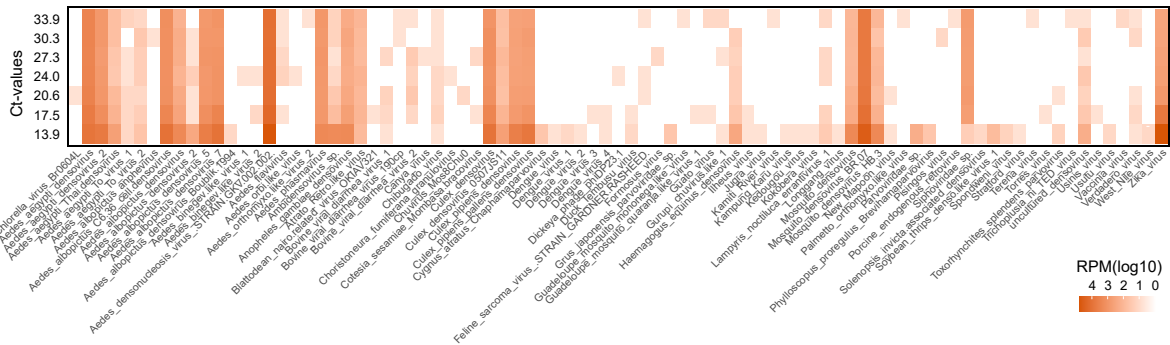

Supplement: Supplementary file 12 — Additional file12: Fig S2. Application of the SMART-RNA-Metavirome platform for detecting viral genomes across various species. A Heatmap representing the RPM of virus detected in JEV isolate with different titers. B Heatmap displaying the RPM of virus detected in ZIKV isolate with different titers. [file 40249_2025_1371_MOESM12_ESM.pdf]
